# Haloperidol dopamine receptor occupancy and antagonism correspond to delirium agitation scores and EPS risk: A PBPK-PD modeling analysis

**DOI:** 10.1177/02698811241309620

**Published:** 2025-01-04

**Authors:** Paul M Burkat

**Affiliations:** Department of Psychiatry, University of Pennsylvania Perelman School of Medicine, Philadelphia, PA, USA

**Keywords:** Antagonism, delirium, haloperidol, occupancy, RASS

## Abstract

**Background:**

Delirium is a severe neuropsychiatric disorder associated with increased morbidity and mortality. Numerous precipitating factors and etiologies merge into the pathophysiology of this condition which can be marked by agitation and psychosis. Judicious use of antipsychotic medications such as intravenous haloperidol reduces these symptoms and distress in critically ill individuals.

**Aims:**

This study aimed to develop a physiologically-based pharmacokinetic (PBPK) model for the antipsychotic medication haloperidol; estimate plasma and unbound interstitial brain concentrations for repetitive haloperidol administrations used in hyperactive delirium treatment; determine dopamine receptor occupancy and antagonism under these conditions; and correlate these results with Richmond Agitation-Sedation Scale (RASS) scores and the risk of developing extrapyramidal symptoms (EPSs).

**Methods:**

The PBPK model for single and repetitive administrations of peroral and intravenous haloperidol was developed with PK-Sim software. The pharmacodynamic (PD) model for RASS scores with haloperidol unbound interstitial brain concentration passed as the regressor was developed with the MonolixSuite 2021R platform.

**Results:**

Peak haloperidol plasma and unbound interstitial brain concentrations following a single 2 mg intravenous dose are 32 ± 5 nM and 2.4 ± 0.4 nM. With repetitive administrations, dopamine receptor occupancy is 70%–83% and D2_L_R antagonism is 1%–10%. Variations in dopamine receptor occupancy correlate with changes in RASS scores in individuals with hyperactive delirium. There is a linear association between the odds ratio of developing EPS and peak D2_L_R antagonism as functions of dopamine receptor occupancy.

**Conclusions:**

Haloperidol dopamine receptor occupancy time course and D2_L_R antagonism parallel RASS score changes and EPS risk, respectively.

## Introduction

According to the American Psychiatric Association’s Diagnostic and Statistical Manual of Mental Disorders, fifth edition (DSM-5), delirium is defined as a disturbance in attention, awareness, and cognition which develops over a short period of time. Delirium occurs at high rates in various clinical environments including intensive care units, emergency departments, hospice units, and post-acute care settings (Elie et al., 2000; Kiely et al., 2003; Lawlor et al., 2000; McNicoll et al., 2003). The electroencephalographic findings in delirium include slowing of posterior dominant rhythms, generalized theta or delta slow-wave activity, and poor organization of background rhythms (Jacobson and Jerrier, 2000; Romano and Engel, 1944). Delirium can be divided into three sub-categories: hyperactive, hypoactive, and mixed (Lipowski, 1983). Perceptual disturbances, such as hallucinations, delusional thought content, and psychomotor agitation, are more prevalent in hyperactive delirium compared to the hypoactive form, which commonly includes somnolence, decreased motor activity, and reduced awareness of surroundings. Factors that are associated with developing delirium include metabolic disturbances, sepsis, organ failure, polypharmacy, and advanced age, among others (Boettger and Breitbart, 2011; Hosker and Ward, 2017). Individuals who experience significant distress secondary to hyperactive delirium symptoms may benefit from short-term use of haloperidol or an atypical antipsychotic medication until they resolve (Devlin et al., 2018). Although haloperidol may not significantly change delirium duration or decrease mortality, it is efficacious for symptom management (Andersen-Ranberg et al., 2023; Girard et al., 2018). The Richmond Agitation-Sedation Scale (RASS) is a reliable and valid 10-point scale used to evaluate agitated behavior in medical settings and monitor medication efficacy (Sessler et al., 2002). Intermittent intravenous haloperidol administration is an approach to treat hyperactive delirium symptoms in critically ill individuals when non-pharmacological interventions are ineffective and stably reduces RASS scores in individuals with agitated delirium receiving palliative care for advanced cancer (Hui et al., 2017).

Since there is marked heterogeneity in the underlying causes of delirium, there are several mechanistic theories hypothesized to explain the phenomenology: neuroinflammatory, neuronal aging, oxidative stress, neurotransmitter abnormalities, neuroendocrine dysfunction, diurnal dysregulation, and network disconnectivity. Psychomotor agitation and psychosis in delirium, as in primary psychotic disorders such as schizophrenia, may be associated with dopaminergic hyperactivity in certain brain regions (Howes et al., 2007, 2009; Maldonado, 2013). It was determined from positron emission tomography (PET) scans that the likelihood of reducing symptoms of psychosis in schizophrenia with haloperidol increases with dopamine receptor occupancy exceeding 65% (Kapur et al., 2000). However, antipsychotic medications can also induce adverse effects such as EPS manifested as movement disorders, including parkinsonism, dystonia, akathisia, and tardive dyskinesia (Siafis et al., 2023). EPS is linked to antipsychotic medication association kinetics with the dopamine 2 receptor (D2R)-binding domain, D2R antagonism, and dopamine receptor occupancy greater than 80% (Casey, 2004; Sykes et al., 2017).

Haloperidol (4-[4-(4-chlorophenyl)-4-hydroxypiperidin-1-yl]-1-(4-fluorophenyl)-butan-1-one) is a butyrophenone neuroleptic first introduced into clinical practice in 1959 (López-Muñoz and Alamo, 2009). It is a potent antagonist of the G-protein-coupled D2R, which inhibits adenylate cyclase in the presence of agonists and triggers a series of intracellular second-messenger cascades (Neve et al., 2004). The D2R extended binding domain for the atypical antipsychotic risperidone was identified, and reanalysis of the D2R-haloperidol complex revealed a second extended binding domain that interacts with haloperidol and is involved in direct agonist activation (Fan et al., 2020; Wang et al., 2018). The D2R exists in short (D2_S_R) and long (D2_L_R) isoforms, created by alternative messenger RNA splicing, that have distinct functions (Giros et al., 1989). D2_S_R serves a presynaptic auto-receptor function, while D2_L_R acts at postsynaptic sites and underlies the cataleptic effects of haloperidol (Usiello et al., 2000).

This study aimed to (1) develop a PBPK model for intravenous haloperidol to predict plasma and unbound interstitial brain concentrations with repetitive administrations as used in the treatment of hyperactive delirium; (2) determine the putative time courses of dopamine receptor occupancy from plasma concentrations and D2_L_R antagonism from unbound interstitial brain concentrations with the PBPK model; and (3) associate these findings with RASS scores in hyperactive delirium and the risk of developing EPS. Pharmacodynamic modeling was used to quantitatively establish the relationship between haloperidol unbound interstitial brain concentrations and RASS scores. It was determined that there are strong correlations between variations in occupancy with RASS score changes and between antagonism and EPS odds ratio as functions of D2R occupancy.

## Methods

### Data selection and analysis

The methods for this study are as previously described (Burkat, 2023). Data and initial parameter values were obtained from the results of PubMed (https://pubmed.ncbi.nlm.nih.gov/), PubChem (https://pubchem.ncbi.nlm.nih.gov/), and DrugBank (https://go.drugbank.com/) searches (Kato et al., 2012; Kudo and Ishizaki, 1999). Individual data points from studies were extracted from the published literature using WebPlotDigitizer 4.5 software (https://apps.automeris.io/wpd/) (RASS scores: (Hui et al., 2017); EPS odds ratio: (Siafis et al., 2023)). Data were analyzed using Microsoft Excel (2021; Microsoft Corp., Redmond, WA, USA) and Origin 2024 (OriginLab Corp., Northampton, MA, USA). Haloperidol D2R occupancy versus plasma concentrations was fitted with the Hill equation *y* = 100*[Haloperidol]^*n*^/(*k*^^*n*^^ + [Haloperidol]^^*n*^^) (*k* = 0.52, *n* = 0.36) (Uchida et al., 2011). Haloperidol concentration-dependent accumulation of cyclic adenosine monophosphate (cAMP, Antagonism) measured from a recombinant D2_L_R in vitro system was fitted with the logistic equation *y* = *A*_2_ + (*A*_1_ − A_2_)/(1 + ([Haloperidol]/EC_50_)^*p*^) (*A*_2_ = 100, *A*_1_ = 0, EC_50_ = 22 nM, *p* = 1.1) (Masri et al., 2008). Linear fits for peak unbound interstitial haloperidol brain concentrations versus peak plasma concentrations, unbound interstitial brain concentrations versus haloperidol intravenous dose, odds ratio of EPS versus peak D2_L_R antagonism, PBPK model evaluation, and PD model evaluation used a weighted least-square method.

### PBPK model development

**Figure 1.**
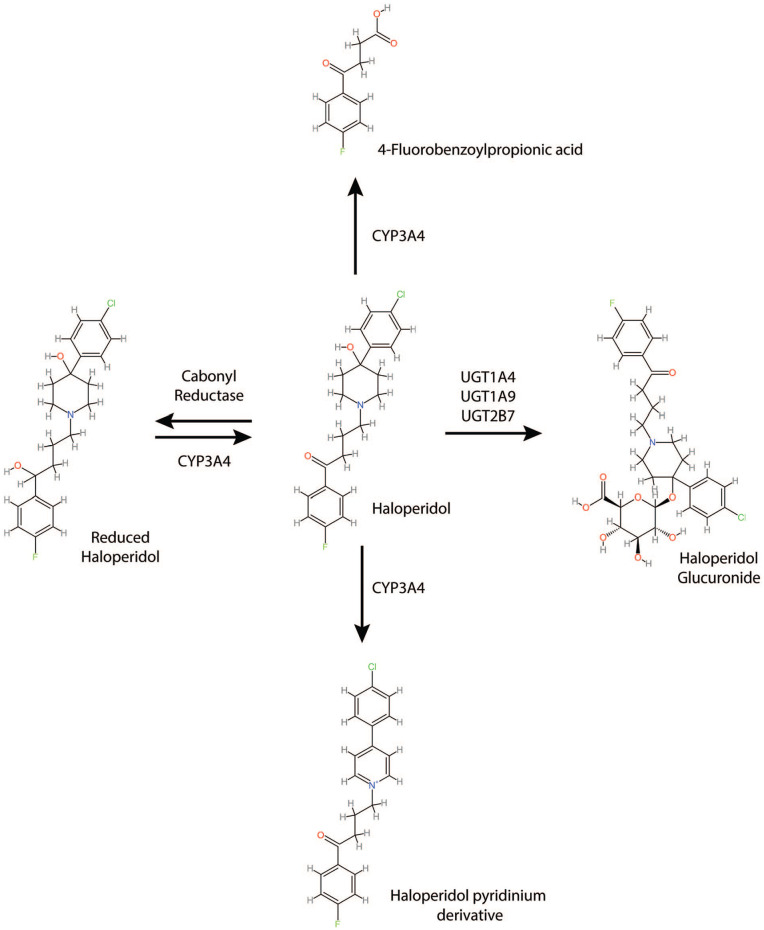
The processes for haloperidol metabolism.

PBPK models can be considered flow- or permeability-limited and are described by systems of differential equations with parameters that include compartment concentrations as functions of time, blood flow rates, compartment volumes, partition coefficients, and permeability-surface area products (Thompson and Beard, 2011). The PBPK model for haloperidol was developed with PK-Sim software (version 11.0, 2023, www.open-systems-pharmacology.org). The virtual individual used for model development was based on a White American male (30 years old, 80.35 kg, 178.5 cm, BMI 25.2). All relevant anatomical and physiological characteristics of an individual from this population are defined in PK-Sim. Population haloperidol plasma and unbound interstitial brain concentration–time profiles were from a group of 100 individuals based on the virtual individual used in model development. This population consisted of 50 females and 50 males (25–55 years old, 55–86 kg, 155–180 cm, BMI 18.5–24.9). Modeling cytochrome P450–drug interactions utilized an associated gene expression database which provides the relative expression of enzymes throughout all organ tissues. Haloperidol metabolism processes were defined in the metabolizing enzyme branch of PK-Sim according to the reaction scheme shown in Figure 1.

The standard organ distribution model in PK-Sim consists of blood cell, plasma, interstitial space, and intracellular space compartments, and considers a permeation barrier between plasma and interstitial space. The rate of permeation through the endothelial barrier is determined by the product of endothelial permeability and surface area. Unbound interstitial brain concentrations can be derived by multiplying the total interstitial space concentration with the water:interstitial partition coefficient. Partition coefficients and cellular permeabilities for tissue distribution were calculated according to the PK-Sim Standard method. The relevant compound parameters for this method are the octanol:water partition coefficient (Log P) and plasma protein binding.

The Rodgers and Rowland method for calculating tissue partition coefficients considers electrostatic interactions between ionized compounds and anionic phospholipids at physiological pH, and interactions with intracellular neutral phospholipids and neutral lipids. Two additional input parameters are necessary for the calculation of tissue partition coefficients: the blood:plasma concentration ratio as a measure of drug electrostatic interactions with acidic phospholipids, and the oil:water partition coefficient which is a better surrogate than Log P for neutral lipid. These parameters are unknown for haloperidol; therefore, the Rodgers and Rowland method was not implemented in this study (Alasmari et al., 2022). The brain interstitial:plasma partition coefficient was optimized to yield an unbound interstitial brain:unbound plasma concentration ratio of 1.54 (Summerfield et al., 2016).

Local parameter sensitivity analysis was performed on all model input parameters prior to parameter identification for optimization. A subset of model parameters was optimized using a Monte Carlo algorithm while fitting the intravenous plasma concentration–time profile (Table 1). For peroral simulations, the confirmed intravenous model was utilized without parameter changes. Tablet dissolution was modeled with a Weibull function with a parabolic shape parameter (0.92), 45 min dissolution time, and zero lag time.

**Table 1.** Haloperidol drug-dependent model parameters.

Parameter	Value	Optimized	Unit	Ref.	Description
Haloperidol					
M.W.	375.86		g/mol	https://go.drugbank.com/drugs/DB00502	Molecular weight
pKa_1_	13.96			https://go.drugbank.com/drugs/DB00502	Acid dissociation constant
pKa_2_	8.05			https://go.drugbank.com/drugs/DB00502	Acid dissociation constant
Log P	3.7	3.63		https://go.drugbank.com/drugs/DB00502	Lipophilicity
Solubility	0.00446		mg/mL	https://go.drugbank.com/drugs/DB00502	Aqueous solubility
f_u_	7.5–11.6	8	%	Kudo and Ishizaki (1999)	Unbound fraction
V_max_ oxidative *N*-dealkylation	180–412	289	pmol min^−1^ mg^−1^	Kudo and Ishizaki (1999)	Maximum reaction velocity
K_m_ oxidative *N*-dealkylation	50–78		µM	Kudo and Ishizaki (1999)	Michaelis–Menten constant
V_max_ carbonyl reduction	1.01–1.11	1.1	nmol min^−1^ mg^−1^	Kudo and Ishizaki (1999)	Maximum reaction velocity
K_m_ carbonyl reduction	500–610		µM	Kudo and Ishizaki (1999)	Michaelis–Menten constant
V_max_ oxidation	0.53		pmol min^−1^ pmol^−1^	Kudo and Ishizaki (1999)	Maximum reaction velocity
K_m_ oxidation	80		µM	Kudo and Ishizaki (1999)	Michaelis–Menten constant
V_max_ back-oxidation of reduced haloperidol	191–334	269	pmol min^−1^ mg^−1^	Kudo and Ishizaki (1999)	Maximum reaction velocity
K_m_ back-oxidation of reduced haloperidol	52–59	55	µM	Kudo and Ishizaki (1999)	Michaelis–Menten constant
V_max_ UGT1A4 glucuronidation	0.6	0.054	nmol min^−1^ mg^−1^	Kato et al. (2012)	Maximum reaction velocity
K_m_ UGT1A4 glucuronidation	64	53	µM	Kato et al. (2012)	Michaelis–Menten constant
V_max_ UGT1A9 glucuronidation	2.3	0.08	nmol min^−1^ mg^−1^	Kato et al. (2012)	Maximum reaction velocity
K_m_ UGT1A9 glucuronidation	174	62	µM	Kato et al. (2012)	Michaelis–Menten constant
V_max_ UGT2B7 glucuronidation	1	0.07	nmol min^−1^ mg^−1^	Kato et al. (2012)	Maximum reaction velocity
K_m_ UGT2B7 glucuronidation	45	21	µM	Kato et al. (2012)	Michaelis–Menten constant
Brain interstitial:plasma	0.4174 (calculated)	430			Partition coefficient

### Pharmacodynamic model development

The haloperidol PD model for a percent decrease in RASS scores associated with the treatment of hyperactive delirium was implemented with the MonolixSuite 2021R platform (Lixoft Corp., Antony, France). Initial goodness-of-fit for indirect PD models was judged by eye, and final goodness-of-fit was determined by linear correlation of observed versus predicted values. Parameters in the PD model were optimized using the Stochastic Approximation Expectation-Maximization (SAEM) algorithm (Table 2; Airoldi and Toulis, 2015). Percent decrease in RASS score with haloperidol unbound interstitial brain concentration passed as the regressor was modeled with the equation:

$$2.\;\frac{dE}{dt}=R_{in}\left(1+\frac{E_{max}+C(t)}{C(t)+C_{50}}\right)-k_{out}*E$$

where *E* is the effect, *R_in_* is the input rate, *E_max_* is the maximal effect, *C*(*t*) is haloperidol unbound interstitial brain concentration as a function of time, *C*_50_ is the concentration where the effect is half maximal, and *k_out_* is the output rate constant.

**Table 2.** Pharmacodynamic model parameters.

Haloperidol	
*E_max_*	25.31
*C*_50_	0.24
*R_in_*	16.66
*k_out_*	2.78

## Results

### Final PBPK model predicts haloperidol intravenous and peroral plasma concentration–time courses

**Figure 2.**
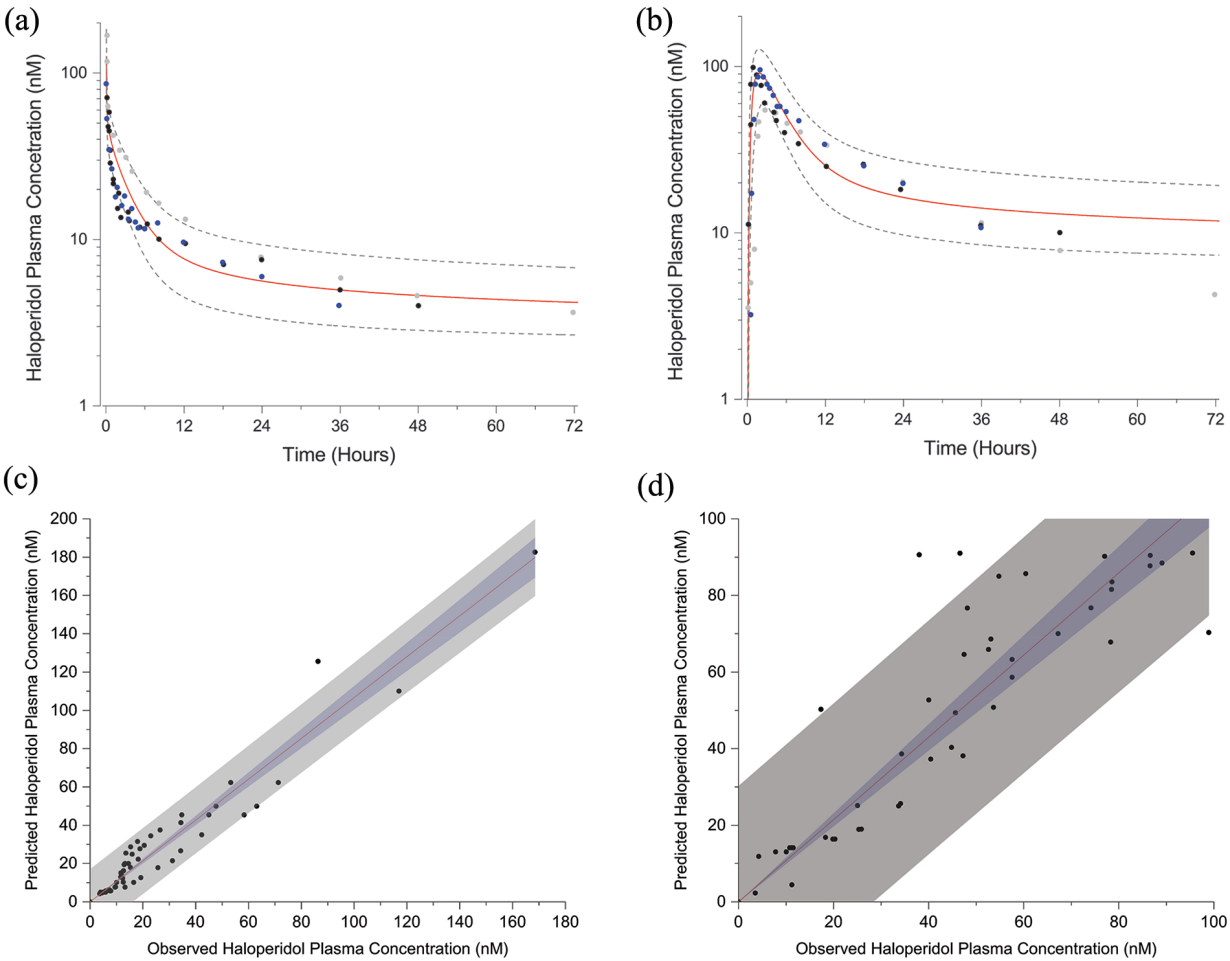
Population simulations of the PBPK model to 0.125 mg/kg intravenous (a) and 0.5 mg/kg peroral (b) administrations of haloperidol (means ± SD). Filled symbols are observed plasma concentrations. Linear regression fits (95% confidence bands (blue regions), 95% prediction bands (gray regions)) to observed versus predicted plasma concentrations for intravenous (c) and peroral (d) administrations of haloperidol. Source: Adapted from Chang et al. (1992), Holley et al. (1983), and Magliozzi et al. (1985).

For PBPK model development, three studies were identified that determined haloperidol plasma concentration–time courses following both 0.125 mg/kg intravenous and 0.5 mg/kg peroral single administrations (Chang et al., 1992; Holley et al., 1983; Magliozzi et al., 1985). The simulated intravenous mean peak plasma concentration is 126 nM (80–183 nM) at 0.7 min, which decreases to half-maximal concentration in 9 min (Figure 2(a)). The simulated peroral mean peak plasma concentration is 91 nM (55–127 nM) at 1.8 h, which decreases to half-maximal concentration at 6.6 h (Figure 2(b)). PBPK model performance was evaluated with linear regression of predicted versus observed haloperidol plasma concentrations. Linear fit parameters for intravenous administration are 1.07 ± 0.03 (slope) and 0.979 (Pearson’s *r*) (*n* = 53) (Figure 2(c)); 1.07 ± 0.04 (slope) and 0.966 (Pearson’s *r*) (*n* = 46) for peroral administration (Figure 2(d)).

### PBPK model predictions of haloperidol concentrations with repetitive administrations

**Figure 3.**
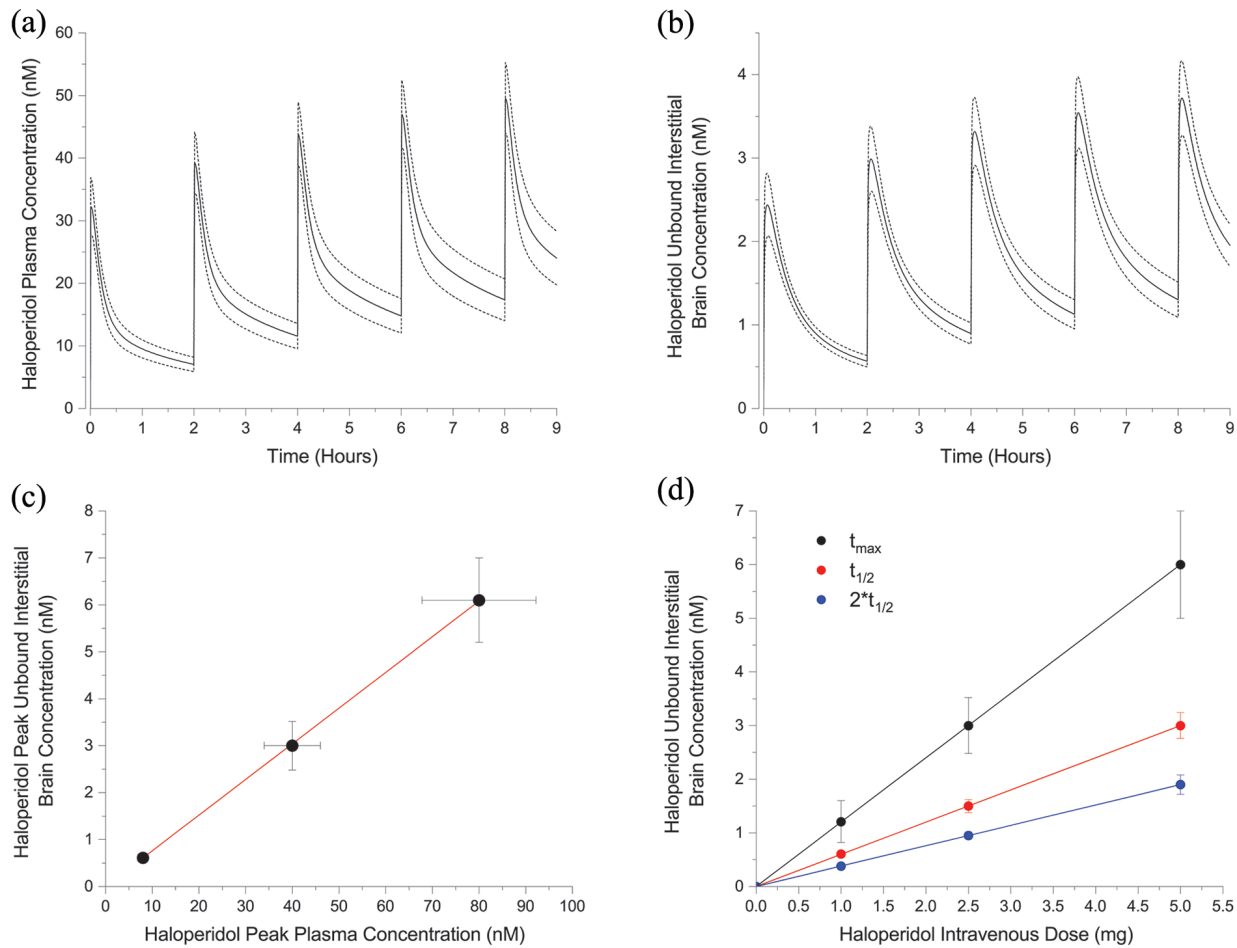
Population simulations of plasma (a) and unbound interstitial brain concentrations (b) following 2 mg intravenous haloperidol administrations administered every 2 h (means ± SD). Haloperidol peak unbound interstitial brain concentrations versus peak plasma concentrations (c) and unbound interstitial brain concentrations versus intravenous administration doses (means ± SD) with linear fits superimposed.

With the confirmed PBPK model, population haloperidol plasma (Figure 3(a)) and unbound interstitial brain (Figure 3(b)) concentrations were simulated for a 2 mg intravenous administration delivered every 2 h over 9 h. Peak plasma concentration is 32 ± 5 nM following the first administration and 49 ± 6 nM following the fifth administration. Peak unbound interstitial brain concentrations following the first and fifth administrations are 2.4 ± 0.4 nM and 3.7 ± 0.4 nM, respectively. Haloperidol demonstrates linear pharmacokinetic properties (Cheng and Paalzow, 1992). Haloperidol unbound brain concentrations increase linearly with plasma concentration (peak brain concentration = 0.076* peak plasma concentration) (Figure 3(c)) and with intravenous dose (peak brain concentration = 1.2 * dose) (Figure 3(d)).

### Haloperidol dopamine receptor occupancy and antagonism associated with RASS scores and EPS

**Figure 4.**
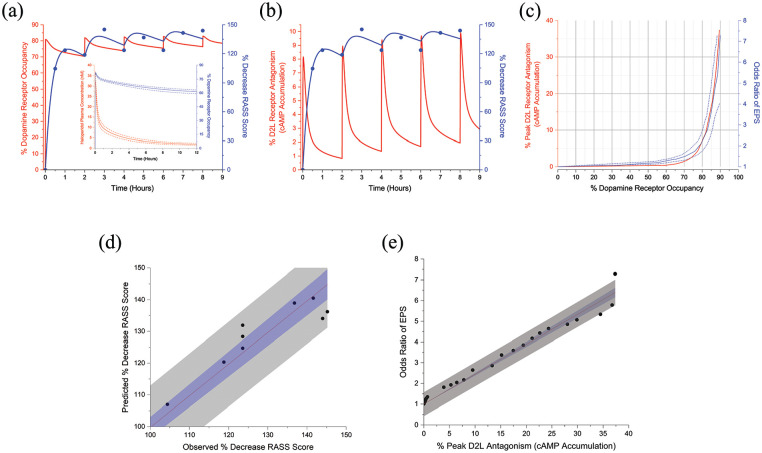
Percent decrease in RASS scores (filled symbols) with pharmacodynamic model responses superimposed (blue lines) associated with dopamine receptor occupancy (a) and antagonism (b) in response to 2 mg intravenous haloperidol administrations delivered every 2 h. Adapted from Hui et al. (2017). Inset, haloperidol plasma concentration and dopamine receptor occupancy versus time following single 2 mg intravenous administration. (c) Peak D2_L_R receptor antagonism (red line) and odds ratio of EPS (solid blue line) versus percent dopamine receptor occupancy. Dashed blue lines represent odds ratio confidence intervals. Adapted from Siafis et al. (2023) (d) Predicted RASS score changes from the pharmacodynamic model versus observed values with a linear fit. (e) Odds ratio of EPS versus percent peak D2_L_R antagonism with linear fit. 95% confidence bands (blue regions) and 95% prediction bands (gray regions).

The simulated plasma concentrations following 2 mg intravenous haloperidol delivered every 2 h from Figure 3(a) were used to estimate dopamine receptor occupancy over 9 h. Mean peak dopamine receptor occupancy following the first administration is 81% which declines to 70% at 2 h. Mean peak occupancy following the fifth administration is 83% and 78% 1 h later. The association between dopamine receptor occupancy and changes in RASS scores is demonstrated in Figure 4(a). Although Hui et. al. concluded that RASS scores were quasi-stable over the haloperidol treatment course, correlation with dopamine receptor occupancy indicates there are apparent quantitative differences with each dose due to concentration and time-dependent occupancy differences (Hui et al., 2017).

D2_L_Rs are coupled to signaling that is mediated by activation of Gα_i/o_ heterotrimeric G-proteins, which causes inhibition of intracellular cAMP accumulation. In the presence of the selective D2-like full agonist quinpirole, which has a receptor affinity nearly equivalent to dopamine, haloperidol is an antagonist of D2_L_Rs leading to an accumulation of cAMP (Bonifazi et al., 2017; Masri et al., 2008; Neve et al., 2004). Haloperidol unbound interstitial brain concentrations from Figure 3(b) were therefore used to determine magnitudes and time course of D2_L_R antagonism with repetitive 2 mg intravenous administrations and then correlated with changes in RASS scores. Peak antagonism is 8% with the first administration, which decreases to approximately 1% at 2 h. Following the fifth administration, peak antagonism is 10% and 3% 1 h later (Figure 4(b)). Pharmacodynamic model performance was evaluated with linear regression of predicted versus observed RASS score changes. Linear fit parameters are 0.997 ± 0.015 (slope) and 0.999 (Pearson’s *r*) (*n* = 9) (Figure 4(d)).

Peak D2_L_R antagonism was determined for unbound interstitial brain concentrations over a series of intravenous haloperidol doses and synchronized with dopamine receptor occupancy from the corresponding plasma concentrations for the same intravenous doses. This function was then associated with the haloperidol odds ratio of developing EPS in human subjects (Figure 4(c)) (Siafis et al., 2023). Peak antagonism is 1% for 65% dopamine receptor occupancy which increases to 7% for approximately 80% occupancy. There is a linear correlation between the odds ratio of EPS and peak D2_L_R antagonism as functions of dopamine receptor occupancy (Slope = 0.144 ± 0.003; Pearson’s *r* = 0.959) (Figure 4(e)).

## Discussion

This study developed a PBPK model for intravenous haloperidol and predicted plasma and unbound interstitial brain concentrations for repetitive administrations as in the treatment of individuals with hyperactive delirium. Variations in dopamine receptor occupancy correspond to changes in RASS scores in these individuals. With 2 mg intravenous haloperidol administrations delivered every 2 h, estimated dopamine receptor occupancy is between 71% and 83% and D2_L_R antagonism is between 1% and 10% over 9 h. Pharmacodynamic modeling quantitatively establishes the association between haloperidol unbound interstitial brain concentrations and RASS scores. Peak D2_L_R antagonism is linearly correlated with the odds ratio of developing EPS. The estimated peak D2_L_R antagonism is 1% for 65% receptor occupancy and 7% for 80% occupancy.

Therapeutic haloperidol plasma concentrations for the treatment of hyperactive delirium are unknown; however, intravenous dose ranges have been suggested depending on symptom severity (0.5–20 mg). Administrations are repeated until symptom improvement is achieved, followed by a maintenance dose that is 25% of the loading dose delivered every 6–12 h as needed. For the treatment of acute psychosis in schizophrenia, haloperidol plasma concentration therapeutic thresholds range from 8–21 nM (lower limit) to 29–69 nM (upper limit) (Ulrich et al., 1998). These therapeutic thresholds correspond to peak D2_L_R antagonism ranging from 2% to 18% based on the analysis in this study. With normal D2R synaptic densities, it has also been suggested that optimal antipsychotic receptor occupancy for acute psychosis treatment is 65%–78% (Iyo et al., 2013).

A central nervous system (CNS) hyperdopaminergic state has been hypothesized to underly several clinical manifestations in some forms of delirium (Mash, 2016). Concentrations of the dopamine metabolite homovanillic acid (HVA) in cerebrospinal fluid (CSF) reflect dopamine turnover in mesolimbic and mesostriatal areas and may be an indirect marker of dopamine pathway functioning in the CNS (Marín-Valencia et al., 2008). Several studies have demonstrated elevated plasma HVA concentrations in individuals with delirium associated with hepatic failure, Alzheimer’s dementia, and other neurological conditions compared to individuals without delirium (Knell et al., 1974; Ramírez-Bermúdez et al., 2019; van der Cammen et al., 2006). In addition, elevated CSF concentrations of HVA are associated with psychotic symptoms in delirium (Ramirez-Bermudez et al., 2008). Mechanisms leading to increased CNS dopamine concentrations under hypoxic conditions include decreased dopamine conversion to norepinephrine, decreased dopamine degradation by cathechol-o-methyltransferase, decreased dopamine re-uptake into presynaptic neuronal terminals, and increased dopamine synthesis (Maldonado, 2013).

Dysfunction of cortico-striato-thalamo-cortical and mesostriatal circuits plays a significant role in the pathogenesis with regard to motor activity and psychosis. Excessive dopamine stimulation of dopamine type 1 receptors in the direct striatal pathway pauses neuronal firing in the globus pallidus internus, which may contribute to hyperkinesia in individuals with hyperactive delirium (Lanciego et al., 2012). Haloperidol, in turn, antagonizes D2Rs, leading to disinhibition of the globus pallidus internus via the indirect striatal pathway, and therefore reduces motor activity (Loonen and Ivanova, 2013). Furthermore, dopamine synthesis, release capacity, and synaptic concentrations are considered to be higher in individuals with primary psychotic disorders compared to healthy controls, and dysregulated striatal dopaminergic signaling contributes to psychotic symptoms (Howes et al., 2024). Mesostriatal dopaminergic neurons modulate signal prediction errors and misperception behaviors which are correlated with false associations (delusions) and hallucinations (Farrell et al., 2022; Schultz et al., 1997; Schmack et al., 2021). Similarly, haloperidol antagonism of D2Rs in these regions alleviates positive symptoms of psychosis. These effects, reduction of motor activity and psychosis, still make antipsychotic medications useful tools in the symptomatic management of hyperactive delirium, albeit at the smallest doses and shortest durations possible (Stollings et al., 2021).

Acute EPS (akathisia, dystonia, parkinsonism), which may occur within a day of starting antipsychotic medications, are dose- and class-dependent. EPS is more prevalent with high-potency, first-generation antipsychotic medications, such as haloperidol, relative to second-generation atypical antipsychotic medications (Dayalu and Chou, 2008; Novick et al., 2010). Of the 15 individuals who received intravenous haloperidol for hyperactive delirium and demonstrated RASS score reductions, an average of 4 developed hypokinesia/akinesia, 2 hyperkinesia, and 1 akathisia (Hui et al., 2017). Other studies have similarly suggested that approximately 30% of individuals with delirium taking 5–15 mg daily doses of haloperidol experience EPS (Zakhary et al., 2024). It is well known that the risk of developing EPS increases when dopamine receptor occupancy is greater than 80% (Farde et al., 1992). This depends on antipsychotic medication D2R-binding affinity. In addition, it was determined that more rapid association, not dissociation, binding kinetics for a range of antipsychotic drugs correlate with EPS risk (Sykes et al., 2017). The findings of this study further suggest a linear relationship between the odds ratio of developing EPS and antagonism of D2_L_Rs by haloperidol, both as functions of dopamine receptor occupancy. Further studies that examine these relationships for other antipsychotic medications will provide additional insight into the mechanisms of drug-induced movement disorders.

Tauscher et al. (2002) established a dissociation between D2R occupancy and plasma pharmacokinetics after single peroral doses of the atypical antipsychotic medications risperidone and olanzapine and suggested that studies of brain kinetics, rather than plasma pharmacokinetics, may provide better guidance for optimizing psychotropic dosing regimens in the treatment of neuropsychiatric disorders. This dissociation can also be demonstrated by utilizing a PBPK modeling approach (Wong et al., 2019). Following a single 2 mg intravenous dose of haloperidol, the calculated peak D2R occupancy is 81%, which declines to 61% at 12 h according to the analysis of this study (Figure 4(a), *inset*). Furthermore, there are regional differences in haloperidol concentrations within brain tissue which indicates mechanisms of uptake across CNS blood–brain and blood–CSF barriers vary spatially (Kornhuber et al., 1999). Loryan et al. further concluded that there are regional differences in CNS unbound antipsychotic extracellular fluid concentrations. K_p,uu,ROI_ ranged from approximately 0.9–1.5 for haloperidol under steady-state conditions, with the highest penetration in the hippocampus, striatum, and frontal cortex (Loryan et al., 2016). The K_p,uu_ utilized in this study was 1.54, which may provide estimates of unbound haloperidol concentrations in these specific areas.

The PBPK and PD models developed in this study utilized a virtual population with parameters determined for healthy individuals, different from populations treated with intravenous haloperidol for hyperactive delirium. The pharmacodynamic responses to drug action are critically dependent on pharmacokinetic characteristics, including absorption, distribution, metabolism, and elimination. Two determinants for PK/PD alterations in critical illness are drug–drug interactions and the pathophysiological changes that occur in patients. Understanding these interactions and changes is essential to optimize drug dosing to achieve pharmacodynamic targets and minimize adverse effects. Some of the pathophysiological changes that occur in critical illness include alterations in pH, fluid shifts, plasma protein binding, blood flow rates, and organ dysfunction, among others (Boucher et al., 2006). Hypoalbuminemia, which is highly prevalent in critically ill individuals, decreases protein-bound drug concentrations and increases the unbound fraction that is free to distribute to target organs possibly leading to adverse effects (Nicholson et al., 2000). Capillary leak and fluid extravasation tend to increase a drug’s volume of distribution, which may lead to subtherapeutic plasma concentrations. Conversely, renal and/or hepatic dysfunction may decrease drug clearance resulting in supratherapeutic plasma concentrations (De Paepe et al., 2002; Jamal et al., 2018; McKindley et al., 1998).

In conclusion, haloperidol dopamine receptor occupancy and peak D2_L_R antagonism are strongly correlated with changes in agitation scores in individuals with hyperactive delirium and the risk of developing EPS. The results of this study may assist in determining optimal drug dosing strategies for hyperactive delirium treatment that minimize adverse effects. Further studies examining the relationships between other antipsychotic medications, occupancy, and antagonism will provide additional insight into the treatment of critically ill patients and the mechanisms of drug-induced movement disorders.
